# Fractional Anisotropy in Corpus Callosum Is Associated with Facilitation of Motor Representation during Ipsilateral Hand Movements

**DOI:** 10.1371/journal.pone.0104218

**Published:** 2014-08-13

**Authors:** Shin-Yi Chiou, Ray-Yau Wang, R. Edward Roberts, Yu-Te Wu, Chia-Feng Lu, Kwong-Kum Liao, Yea-Ru Yang

**Affiliations:** 1 Department of Physical Therapy and Assistive Technology, National Yang-Ming University, Taipei, Taiwan; 2 Academic Department of Neuro-otology, Charing Cross Hospital Campus, Imperial College London, London, United Kingdom; 3 Department of Biomedical Imaging and Radiological Sciences, National Yang-Ming University, Taipei, Taiwan; 4 Department of Education and Research, Taipei City Hospital, Taipei, Taiwan; 5 Department of Neurology, Taipei Veterans General Hospital, Taipei, Taiwan; University Medical Center Goettingen, Germany

## Abstract

**Background:**

Coactivation of primary motor cortex ipsilateral to a unilateral movement (M1_ipsilateral_) has been observed, and the magnitude of activation is influenced by the contracting muscles. It has been suggested that the microstructural integrity of the callosal motor fibers (CMFs) connecting M1 regions may reflect the observed response. However, the association between the structural connectivity of CMFs and functional changes in M1_ipsilateral_ remains unclear. The purpose of this study was to investigate the relationship between functional changes within M1_ipsilateral_ during unilateral arm or leg movements and the microstructure of the CMFs connecting both homotopic representations (arm or leg).

**Methods:**

Transcranial magnetic stimulation was used to assess changes in motor evoked potentials (MEP) in an arm muscle during unilateral movements compared to rest in fifteen healthy adults. Functional magnetic resonance imaging was then used to identify regions of M1 associated with either arm or leg movements. Diffusion-weighted imaging data was acquired to generate CMFs for arm and leg areas using the areas of activation from the functional imaging as seed masks. Individual values of regional fractional anisotropy (FA) of arm and leg CMFs was then calculated by examining the overlap between CMFs and a standard atlas of corpus callosum.

**Results:**

The change in the MEP was significantly larger in the arm movement compared to the leg movement. Additionally, regression analysis revealed that FA in the arm CMFs was positively correlated with the change in MEP during arm movement, whereas a negative correlation was observed during the leg movement. However, there was no significant relationship between FA in the leg CMF and the change in MEP during the movements.

**Conclusions:**

These findings suggest that individual differences in interhemispheric structural connectivity may be used to explain a homologous muscle-dominant effect within M1_ipsilateral_ hand representation during unilateral movement with topographical specificity.

## Introduction

Primary motor cortex (M1) is involved in motor execution and exerts control over contralateral voluntary movements. However, coactivation of M1 ipsilateral to a limb movement (M1_ipsilateral_) also occurs on the respective homotopic (e.g. right arm movement activates right hemisphere arm area) and heterotopic (right arm movement activates right hemisphere leg area) representations, which are not directly involved in the executed movement [1]–[3]. Transcranial magnetic stimulation (TMS) studies in humans have shown that corticomotor excitability of M1_ipsilateral_ is enhanced during unilateral movement [4]–[9]. The current consensus in the literature is that the transcallosal pathway between bilateral M1s may play an important role in mediating the observed changes in M1_ipsilateral_ excitability. This is supported by TMS studies which report changes in interhemispheric inhibition from contralateral to ipsilateral M1 during unilateral hand movement [10]–[13].

Muscle contraction during a unilateral motor task, especially the muscle homologous to the contralateral muscle of upper limb, can influence facilitation of M1_ipsilateral_ activity [3], [6], [13], [14]. It has been suggested that facilitation of corticospinal excitability in the homotopic representations of M1_ipsilateral_ may be modulated by the microstructure of the interhemispheric pathways linking the two regions. Nevertheless, there has been little research into the relationship between corticospinal excitability of the homotopic representation in M1_ipsilateral_ and interhemispheric structural connectivity. Activity of M1_ipsilateral_ hand representation has been shown to be enhanced by unilateral movement conducted not only via a homologous muscle, but also via a heterologous muscle [3], [8], [9]. This raises the question of whether facilitation of the heterotopic representation is also mediated via interhemispheric structural connectivity or is mediated by ipsilateral corticospinal pathway [15].

The fibers connecting the M1 regions of the two hemispheres are termed callosal motor fibers (CMFs). The CMFs of humans have previously been identified using diffusion tensor imaging (DTI), a technique used to infer properties of the underlying white matter microstructure by quantifying the directionality of diffusion within a voxel by the fractional anisotropy (FA) index [16]. This measure provides an estimate of the local structural coherence of white matter fiber bundles [16]–[20], and in specific brain pathways has frequently been linked to individual differences in performance [21]–[27]. Regional FA has been associated with both interhemispheric function of bilateral M1s and the trans-synaptic excitability of corticospinal output neurons [28], [29]. Therefore, the structural connectivity of the CMFs may also modulate functional changes in M1.

In the present study, we aimed to investigate the relationship between the functional changes within M1_ipsilateral_ during unilateral movements and the microstructure of the CMFs connecting both homotopic representations (arm or leg) in humans. Motor evoked potentials (MEPs) were measured using transcranial magnetic stimulation (TMS) since this parameter is constant and highly sensitive to behavioral settings [2], [3], [8], [12]. The changes of MEPs within M1_ipsilateral_ during unilateral movements compared to rest were calculated and correlated with the mean FA values in the CMFs. We hypothesized that individual differences in the mean level of FA in the CMFs would predict the changes of M1_ipsilateral_ activity induced by contractions of the homologous and heterologous muscles.

## Methods

### Participants

Fifteen right-handed healthy volunteers (eight male) with an average age of 24.8 years (SD: 2.48) participated in the study. The subjects were recruited from the post-graduate students at National Yang-Ming University with similar years of education and all of them took part in both TMS and magnetic resonance imaging (MRI) experiments on different days with the interval no longer than 10 days; the order of TMS and fMRI was counterbalanced across participants. All participants gave written informed consent. The experimental procedures were approved by the Institutional Review Board, Taipei Veterans General Hospital as well as National Yang-Ming University and were performed according to the ethical standards laid down in the Declaration of Helsinki.

### TMS experiment

#### Task

Subjects were seated on the examining bed with hip flexing at 100°. Pillows were placed below the knees and behind the back to support the torso. The participants were required to activate ‘task’ muscles on their right side, the flexor carpi radialis (FCR) of the arm and tibialis anterior (TA) of the leg, while keeping the ‘target’ muscle of the left side, the FCR muscle, relaxed. The experiment comprised two conditions, rest and active. In the rest condition, subjects were instructed to relax and fixate a visual target directly in front of them. In the active condition, subjects were asked to unilaterally activate specific task muscles by forceful tonic contraction while keeping the target muscle on the corresponding side relaxed. The electromyography (EMG) activities of the target and task muscles were displayed on the screen to give feedback to both the participant and the experimenter. The advantage of using the FCR muscle is that there are fewer occurrences of mirror movements and more reliable responses for TMS [30]. There were two pairs of active conditions: 1) right FCR contraction (FCR task) with the left FCR relaxed and 2) right TA contraction (TA task) with the left FCR relaxed. For the FCR task, the elbow was placed at 90° flexion with the forearm and wrist in the neutral position on the pillow. In the active condition, subjects flexed the wrist through the full range of motion. For the TA task, subjects dorsiflexed their right ankle from slight plantarflexion to full dorsiflexion. The two active conditions were applied in a randomized order following the rest condition. The TMS stimulus was delivered 100 ms after the rectified EMG activity reached 75% of maximal EMG activity of each muscle for receiving the optimal facilitating effect [31]; a minimum 5 s break was given between each contraction. In individual traces, background EMG activity on the target muscles was analyzed at 40 ms prior to the onset of TMS stimulus. Trials in which the activity of the target muscles exceeded a background noise level of 25 µV were excluded from analysis [8].

#### Electromyographic recording

Surface electrodes were positioned on the skin overlying both target and task muscles with an active lead positioned on the muscle bellies and a reference lead 4 cm below the active lead. The ground electrode was placed on the left forearm. The sampling rate of the EMG signals was 4 kHz and the signals were amplified with filters set at 20 Hz to 3 kHz and recorded on a computer (Neuropack MEB-9100; Nihon Kohden Corp., Tokyo, Japan).

#### TMS measurements

**Figure 1:**
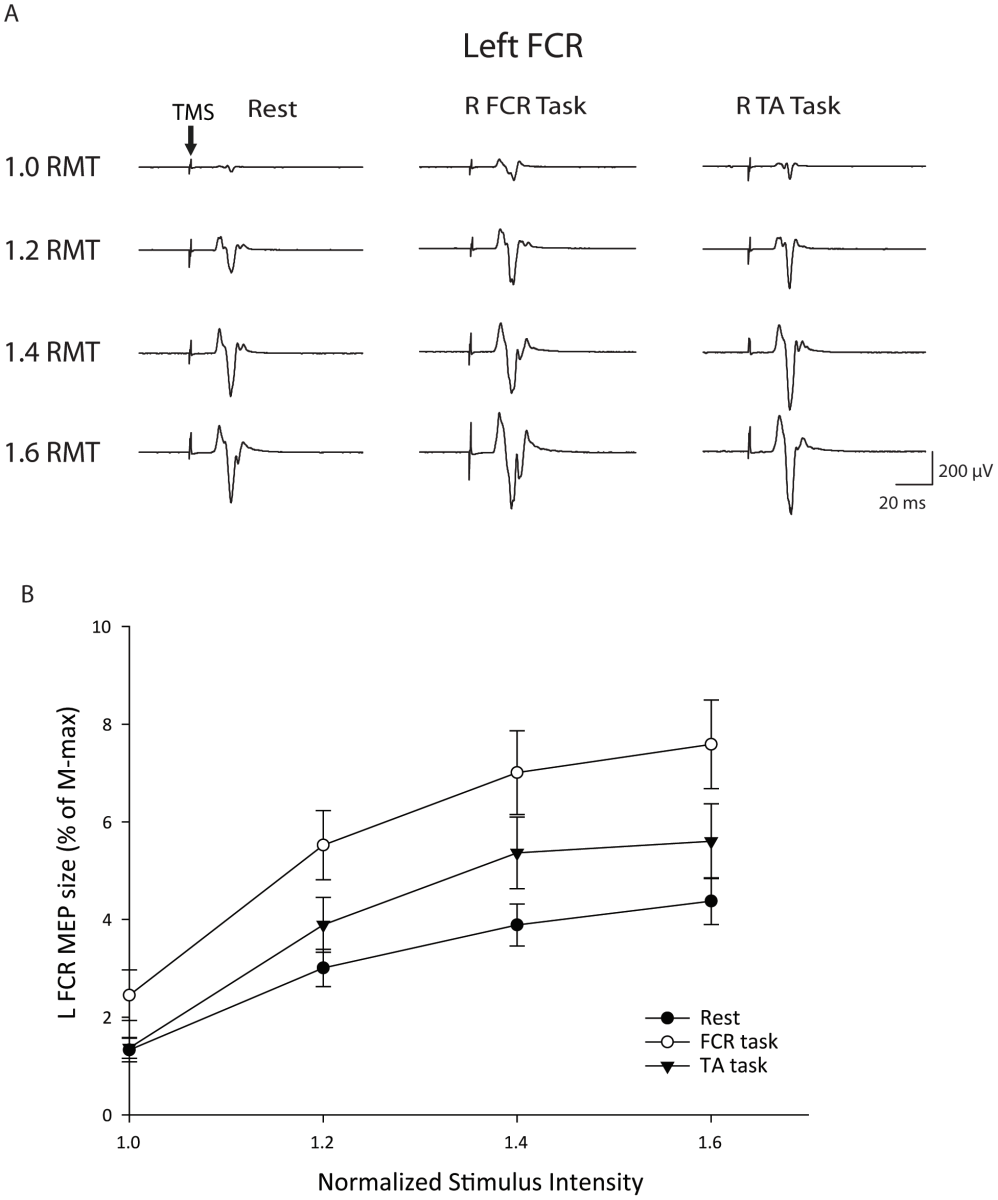
Recruitment curves in the left flexor carpi radialis (FCR). (A) The averaged MEPs recorded from a representative subject. (B) Group data (n = 15) during performance of different motor tasks with right side limbs. The abscissa shows intensity of transcranial magnetic stimulus (TMS) expressed relative to the resting motor threshold (RMT) in each subject. The ordinate shows MEP amplitudes as a percentage of the left FCR M-max. Group data are presented as the mean ± standard error. Arrows indicate delivery times of TMS. TA: tibialis anterior; R: right; L: left.

During the motor task, single-pulse TMS was applied to right M1 using a Magstim 200 magnetic stimulator (The Magstim Company Limited, Spring Gardens, Whitland, Carmarthenshire, UK). A figure-of eight coil (70-mm coil diameter) was used for stimulating the FCR area. The TMS stimulus was directed at the position on M1 which elicited maximal MEP response in the left FCR muscle as the MEPs are highly reliable in the FCR muscle using the standardized function-guided procedure [30]. A swimming cap was used to record the position of the coil, allowing re-positioning of the coil throughout the experiments. The figure-of eight coil was placed over the FCR area of right M1, with the handle pointing backwards and 45° away from the midline. Recruitment curves of MEPs (MEP RCs) were measured in the left FCR muscle while the right task muscles were at rest, and also during the active conditions (Figure 1). Stimulus intensities started at the resting motor threshold (RMT), defined as the lowest intensity of TMS output required to evoke MEPs of at least 50 µV in peak-to-peak amplitude in at least three of five consecutive trials [32], and then increased gradually from 1.2 to 1.8 RMT in steps of 0.2. The averaged RMT of the left FCR was 53.87±6.22% maximal output (range: 42∼63%). Since only 4 out of 15 subjects could have intensity below 100% maximal output at 1.8 RMT (above the maximal output of the machine), MEP amplitudes at this intensity were not included in the further comparison. According to a previous report [33]–[35], a mean of five recorded MEPs resulted in good-to-high reliability in amplitude measures when a single hotspot technique was applied; five MEPs were therefore recorded at each stimulus intensity to avoid general fatigue in the participants. Subjects were allowed to rest between trials in order to avoid muscle fatigue. A maximal motor response (M-max) was collected by stimulating the median nerve (1 ms rectangular pulse) with supramaximal intensity using bipolar surface electrodes in order to normalize the individual MEP amplitudes. Peak-to-peak amplitudes of MEPs were then measured, normalized to the M-max, and averaged off-line. The mean ± standard error (SE) was used to present values of MEP RCs at both rest and active conditions. The change of MEP at each intensity was calculated as the ratio of MEP amplitude between active and rest conditions: (active condition/rest condition) ×100 (%).

### Imaging acquisition

All magnetic resonance images were acquired on a Siemens Trio 3.0T scanner (MAGNETOM Trio, A Tim System 3T, Siemens, Germany) with a 12-channel head coil. Each participant's head was immobilized with cushions inside the coil after alignment to avoid motion artifacts during scanning. Structural 3D T1-weighted data were acquired using a magnetization-prepared rapid-acquisition gradient echo sequence. The imaging parameters were: repetition time (TR)  = 2530 ms, echo time (TE)  = 3.03 ms, flip angle  = 7°, field of view (FOV)  = 224×256×192 mm^3^, voxel size  = 1×1×1 mm^3^. For functional magnetic resonance imaging (fMRI), blood-oxygen-level dependent images of the whole brain were acquired in 40 contiguous axial slices using an echo planar imaging pulse sequence with the following parameters: TR  = 2000 ms, TE  = 20 ms, FOV  = 220×220 mm^2^, slice thickness  = 3.4 mm, and flip angle  = 90°, interleaved acquisition. Subjects performed the same tasks as described in the TMS experiment (FCR task and TA task), with the right side limb. A cushion was placed under the knees to support the body and minimize the translation of movements during the motor tasks. Instructions were presented on a video screen. A total of 328 scans were acquired while subjects performed two blocks of alternating rest and active (16 s per condition and 20 blocks in total). During the active block, the fixation cross alternated between red and white at a rate of 1 Hz; the subjects were asked to contract the muscle once they saw the red cross and to relax the muscle when the white cross appeared.

For the diffusion-weighted imaging data, three acquisitions of thirty-one diffusion-weighted volumes were obtained for each participant, including 30 volumes with diffusion gradients applied along 30 independent orientations (b = 900 s/mm^2^) and 1 volume without diffusion weighting (b0). These were averaged in order to improve the signal to noise ratio of the data. Each volume consisted of 70 continuous axial slices with 2 mm slice thickness covering the entire hemisphere and brainstem using a single shot spin-echo echo planar imaging (EPI) sequence (TR  = 7,900 ms, TE  = 79 ms, number of excitation  = 3, field of view  = 256×256 mm^2^, matrix size  = 128×128).

### fMRI data processing

Off-line fMRI data processing was performed using Statistical Parametric Mapping 8 (http://www.fil.ion.ucl.ac.uk/spm). Correction of slice timing followed by the realignment was applied to all volumes. Functional images were co-registered with T1-weighted anatomical scans for each subject prior to spatial normalization to the standard T1-weighted template of the Montreal Neurological Institute (MNI, Montreal, QC, Canada). Finally, the images were smoothed using an 8-mm full-width-at-half-maximum (FWHM) Gaussian kernel to reduce the effects of noised on the normalized fMRI data. Following spatial normalization and smoothing, statistical analysis was performed. First level mass-univariate analysis primarily modeled the neural response during active and rest blocks with two orthogonal regressors. These regressors were boxcar functions convolved with a canonical hemodynamic response function [36]. Head movements were modeled as nuisance variables with 6 parameter estimates (3 translations and 3 rotations) derived from the realignment process [37]. The mean head motion in 3D space for each brain volume were computed as the root-mean-square of the translation parameters (displacement  =  square root (x^2^+y^2^+z^2^)) and expressed in mm [38]. An F-contrast image of active block (*p*<0.001, uncorrected) was obtained in individual subjects.

Four regions of interests (ROIs) were selected including bilateral FCR representations and TA representations. Coordinates for each ROI were identified in each hemisphere using peak coordinates of task-related activations and confirmed as primary motor cortex using known anatomical labeling [xjview toolbox, (http://www.alivelearn.net/xjview)]. The coordinates of the left FCR representation and that of right FCR representation were (−34, −32, 60 mm) and (40, −22, 58), respectively (MNI). The coordinates of the left TA representation that of right TA representation were (−4, −32, 70) and (4, −30, 66). Here, the fMRI data was only used for identifying locations of the seed masks for tractography (see the *Fiber tracking* section).

### Fiber tracking

FA values were calculated using FMRIB's Diffusion Toolbox (FDT, http://www.fmrib.ox.ac.uk/fsl). Diffusion data were corrected for eddy currents and head motion by using affine registration to a reference volume [39]. Data from the three acquisitions was averaged to improve the signal-to-noise ratio. Probability distributions on fiber direction were calculated at each voxel by using previously described methods [40].

**Figure 2:**
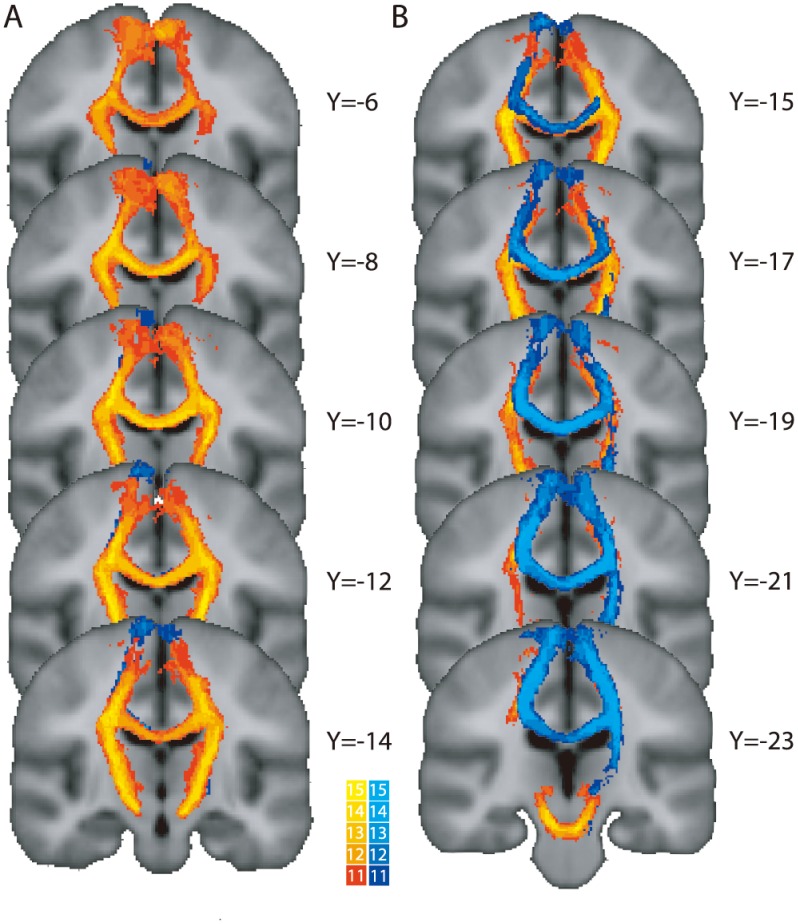
Overlap images for the tracked fibers of hand (A) and leg (B) areas for all subjects superimposed on the Montreal Neurological Institute 152 T1 brain in FSL. The fibers associated with the hand representations are shown in red; the fibers associated with the leg representations are shown in blue. The colour bars indicate the degree of overlap across subjects for each pathway. The sagittal coordinates (Y) are given in standard space (mm) and the image is displayed in the radiological convention.

The coordinates of the peak activations for each muscle group obtained from the fMRI activations were translated into DTI space for each subject and used to create seed masks for tractography; this combined fMRI and DTI approach has been validated in the previous studies [28], [41], [42]. Since the BOLD signal is largely limited to the gray matter, we shifted the position of the seed masks directly towards the nearest white matter tract, rather than enlarge the masks, in order to maintain the specificity of the connections as the seed regions were in close proximity. We considered dilating the seed masks; however, the risk with this approach was the inclusion of fibers from different motor representations distorting the reconstruction of the pathway. Therefore, the FCR seed mask was shifted by six voxels medially and inferiorly, and the TA seed mask was shifted by two voxels laterally (to avoid overlapping ROIs of bilateral TA) and six voxels inferiorly. The new coordinates of the left FCR representation and that of right FCR representation were (−22, −32, 48 mm) and (28, −22, 46), respectively (MNI coordinates); the coordinates of the left TA representation that of right TA representation were (−8, −32, 58) and (8, −30, 54). The seed masks were 8 mm diameter spheres centered on the seed coordinate in order to overlap with sufficient white matter fibers to reconstruct the pathway. The masks were then used to generate probabilistic tractographic paths between the left and right hemisphere regions for FCR and TA representations [43], [44]. The probabilistic maps were thresholded to include only those voxels which had at least 10% of total samples passing through them in order to ensure only connecting fibers were included. Finally, a MNI atlas mask of the corpus callosum was transformed to standard space and overlapped with the tractography paths for both muscle representations (Figure 2). The mean value of FA was calculated for each participant based on the area where the tracked fibers passed through the mask.

### Statistical analysis

We analyzed the data using the statistical package SPSS (version 17.0 for Windows, Chicago, IL). Two-way repeated-measures ANOVA following by a post hoc contrast test was used to determine the effect of CONDITION (rest, FCR task, and TA task) and stimulus INTENSITY (1.0, 1.2, 1.4, and 1.6 times the RMT) on MEP RCs and on background EMG activity to examine the increase of M1_ipsilateral_ activity in the tasks. To investigate the relationship between the mean FA values in the CMFs and the change in MEPs recorded from the left target muscle during the active conditions, stepwise linear regression analysis was employed for the FCR CMFs and TA CMFs separately with the same parameters, the change in MEPs during FCR task and TA task recorded from the left FCR. Age and sex were included as covariates of no interest in the regression analysis. Statistical significance was set at *p*<0.05. Since the two interhemispheric white matter pathways for FCR and TA CMFs occupy neighbouring anatomical areas, we performed a Pearson correlation coefficient analysis of the mean FA values of both FCR and TA CMFs to check whether the FA values in the two tracts were statistically different. Finally, since head motion can be a potential confound in task-based functional MRI studies, we compared the mean head motion during the FCR and TA fMRI tasks using a paired-*t* test.

## Results

### Effect of the unilateral movement on M1_ipsilateral_ activity

Results showed that there was an effect of INTENSITY (F_1.36, 19.00_ = 36.64; *p*<0.001), and CONDITION (F_2, 28_ = 22.18; *p*<0.001), and their interaction CONDITION × INTENSITY (F_2.30, 32.29_ = 3.51; *p* = 0.036) on MEP RCs of the left FCR (Figure 1B). A post hoc contrast test showed that MEP amplitudes at 1.2 times (*p*<0.001), 1.4 times (*p*<0.004), and 1.6 times (*p*<0.003) RMT were greater than that at 1.0 times RMT. Additionally, the post hoc contrast test revealed a significant increase in MEP amplitude in the FCR task (*p*<0.001) and TA task (*p* = 0.016) compared with the rest condition on the left FCR. Furthermore, the post hoc test demonstrated a significant increase in the FCR task compared with the TA task on the left FCR (*p*<0.001; Figure 1B).

There were no significant main effect of INTENSITY (F_1.62, 27.71_ = 1.84; *p* = 0.65), and CONDITION (F_1.06, 14.85_ = 1.41; *p* = 0.26), and their interaction CONDITION × INTENSITY (F_2.47, 34.58_ = 2.16; *p* = 0.12) on background EMG activity of the left FCR muscle.

### Regression analyses of the facilitatory ratios in M1_ipsilateral_ representation and the callosal motor fibers FA values

**Figure 3:**
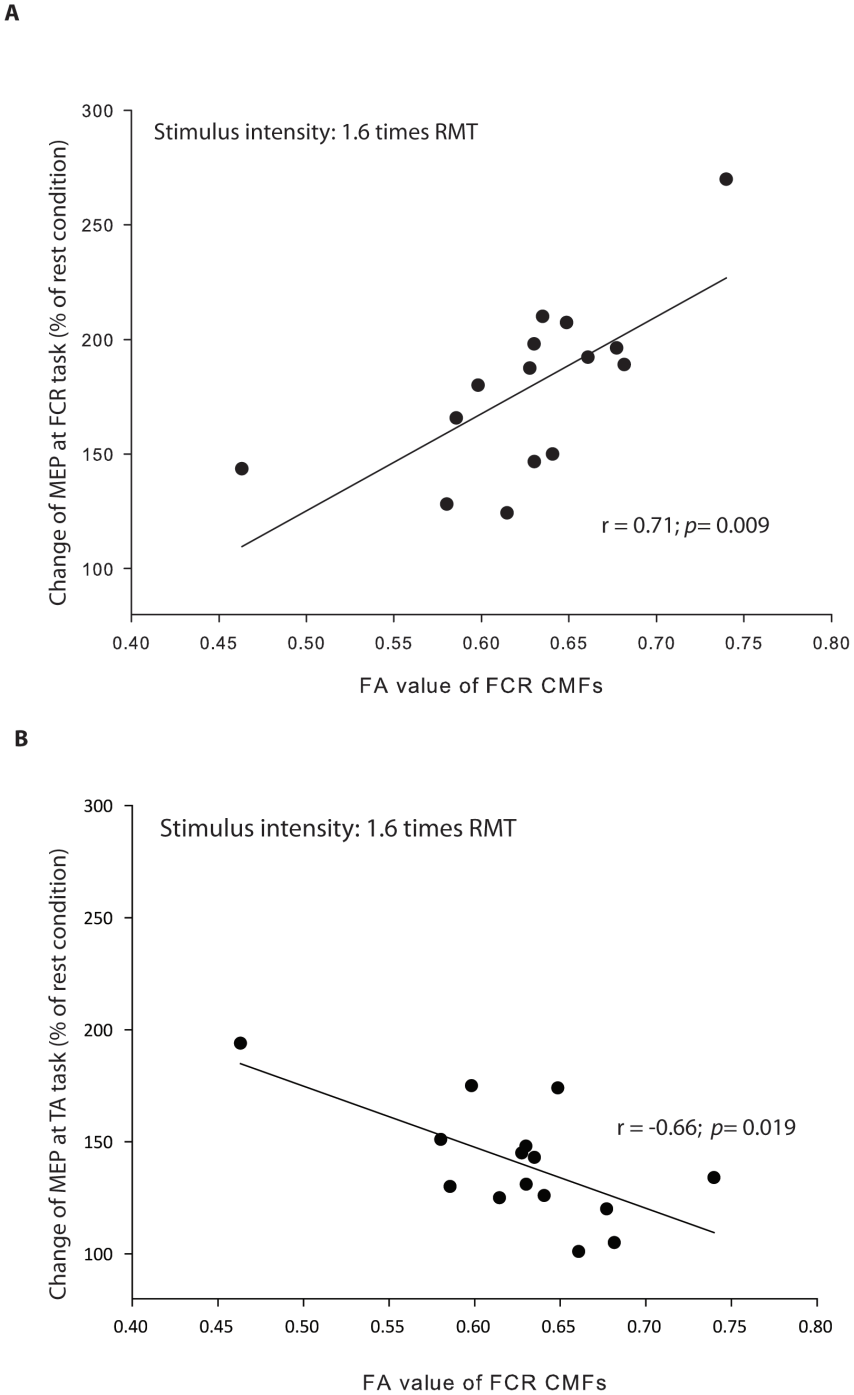
Linkage of callosal motor fibers (CMFs) with functional changes in M1_ipsilateral_ representation during unilateral movement. The fractional anisotropy (FA) of the FCR CMFs is associated with the change of MEP at 1.6 times RMT in (A) right wrist flexion and in (B) right ankle dorsiflexion.

The linear regression analysis revealed a significant positive correlation between FA in the FCR CMFs and the change of MEP at 1.6 RMT recorded during the FCR task (*r* = 0.71; *p* = 0.009), whereas a negative correlation was observed at 1.6 RMT during the TA task (*r* = −0.66; *p* = 0.019) (Figure 3). However, the changes of MEP at the other intensities were not significantly correlated with the mean FA values of FCR CMFs, and were therefore removed from the regression model. Additionally, we detected no significant relationship between FA in the TA callosal motor fibers and the changes of MEPs in left FCR.

There was no significant correlation between the mean values of FCR and TA CMFs (t(14) = −1.671; *p* = 0.12), indicating that there was unlikely to be any significant overlap between the pathways in the tractography reconstruction. Furthermore, mean head motion during the fMRI acquisition was not significantly different for the FCR (0.38±0.11 mm) and TA tasks (0.38±0.15 mm) across subjects (t(14) = −0.013; *p*>0.99), suggesting that there was no significant difference in head motion during the two localization tasks.

## Discussion

In this study we report a significant association between an index of white matter microstructure (FA) and functional change (facilitation of corticospinal excitability) in M1_ipsilateral_ when performing a unilateral motor task. These results reveal that facilitation of the homotopic representation in M1_ipsilateral_ is associated with microstructure in the relevant CMFs during a hand movement, and that FA in the FCR CMFs is associated with reduced facilitation on homotopic M1_ipsilateral_ representation in leg movements performed by the heterologous muscle. Furthermore, the data also suggest that functional changes in the homotopic M1_ipsilateral_ representation may be modulated via representation-specific CMFs.

It has been well documented that corticospinal excitability in a rest muscle of the upper limb can be enhanced by movement of a homologous muscle on the opposite side [3], [6], [13], [14]. We show for the first time that individuals with higher FA values in the fibers connecting FCR regions (and likely denser hand CMFs) had greater facilitation in M1_ipsilateral_ during unilateral movement with homologous muscle. In addition, as a negative correlation was found between FA values and the changes of MEPs in FCR representation in the leg movement, this may reflect a homologous muscle-dominant effect on facilitation of M1_ipsilateral_ reported from previous findings [6], [9], [13].

The biological basis of the FA measurement is still not entirely understood. It has been shown that this measure can be influenced by the degree of myelination, axon size, and axon density [16]–[18]. Light- and electron-microscopic analysis of the fiber composition in the human corpus callosum has demonstrated regional differences, with larger-diameter, myelinated and less densely packed fibers concentrated in the posterior mid-body of the corpus callosum [45], which is the CMF region in humans [28], [46], [47]. This regional differentiation of fiber types and densities is reflected in regional differences in FA, with higher values in areas where the fibers are more densely arranged [47]. It is generally thought that FA in the mid-body of the corpus callosum may primarily reflect fiber density rather than the degree of myelination or axon diameter. Therefore, the association we observed between FA and the change of MEPs in ipsilateral M1 during unilateral movement suggests that more densely packed CMFs could result in a more effective influence on M1_ipsilateral_ activation.

The present finding provides another TMS parameter relevant to the microstructure of bilateral M1s, as interhemispheric inhibition has also been reported as a sensitive marker of microstructural connectivity [28]; it is also in line with previous work, where facilitation of M1_ipsilateral_ during unilateral movements was associated with the change in interhemispheric inhibition from contralateral M1 to ipsilateral M1 [3], [12].

These results are pertinent to the notion that the functional changes due to homologous muscle-dominant effects may be modulated via transcallosal pathways. Further, either the facilitatory effect or the suppression effect on M1_ipsilateral_ is modulated via the representation-specific CMFs, with the higher indices of CMF microstructural properties, the stronger the suppressed/facilitatory effect.

These data also suggest an interesting possibility: there appears to be no general path for contralateral M1 to modulate the activation of M1_ipsilateral_. Instead, the modulation from contralateral M1 to ipsilateral M1 appears to rely on the connection with the targeting muscle itself. This is compatible with the previous finding that the relationship between FA and interhemispheric inhibition was also topographically specific [28].

In this study we only detected significant modulation of interhemispheric connectivity on corticospinal excitability in M1_ipsilateral_ during unilateral movement using a high stimulus intensity. The intensity effect has also been observed in a previous study where a positive association between FA of the hand CMFs and the magnitude of interhemispheric inhibition was reported, but only when intensities of the conditioning pulse were 130% RMT or above [28]. Since the relationship between the connectivity of white matter fibers and the neurophysiology of MEP is not fully understood, this may explain the current discrepancy in results regarding the relationship between TMS paradigms and white matter microstructure [29], [48].

Although these data suggest a modulatory relationship between white matter in CMFs and function changes in M1_ipsilateral_, there are still some limitations to the current study. Since the coordinates of the seed masks had to be slightly adjusted with respect to the activations found in the fMRI task, it may be possible that some white matter fibers in FCR and TA representations were not included in the analysis, and the relationship between CMFs and the changes of MEPs in M1_ipsilateral_ could therefore be diluted. However, by using a combined fMRI and DTI probabilistic tractography approach, we functionally located the seek masks closer to actual functional motor area and included only those tracks whose fibers connected both homotopic representations and passed through corpus callosum [28], [41], [42]. In addition, we did not find any significant correlation between the arm and leg CMFs, indicating that these pathways were unlikely to have overlapped.

The present study provides evidence to link white matter microstructure in the corpus callosum and functional changes in M1 and suggests that interhemispheric structural connectivity may modulate other task-dependent adaptations in M1 ipsilateral to an active muscle. Furthermore, the association between structural connectivity and functional changes in motor cortices may yield opportunities for the development of therapeutic interventions, such as tDCS, to improve impaired motor function by modulating interhemipsheric activity. Further it may pave the way for improved prognostic indicators to assess extent of dysfunction and recovery.
